# Association between prognostic nutritional index and all-cause mortality in critically ill patients with ventilator-associated pneumonia: a retrospective study based on MIMIC-IV database

**DOI:** 10.3389/fnut.2025.1605032

**Published:** 2025-08-18

**Authors:** Wenwen Ji, Guangdong Wang, Jia Liu

**Affiliations:** ^1^Department of Respiratory and Critical Care Medicine, First Affiliated Hospital of Xi’an Jiaotong University, Xi’an, China; ^2^Department of Respiratory Medicine, Honghui Hospital, Xi’an Jiaotong University, Xi’an, China

**Keywords:** prognostic nutritional index, ventilator-associated pneumonia, mortality, cox regression model, restricted cubic spline analysis

## Abstract

**Background:**

Ventilator-associated pneumonia (VAP) remains a significant clinical challenge in the ICU due to its high mortality rate. The Prognostic Nutritional Index (PNI), a composite biomarker based on serum albumin levels and total lymphocyte counts, reflects nutritional and immune status, but its prognostic significance in VAP patients remains unclear. This study evaluated the association between PNI and mortality in critically ill patients with VAP.

**Methods:**

We retrospectively analyzed data from 1,457 patients diagnosed with VAP from the Medical Information Mart for Intensive Care IV (MIMIC-IV) database. Patients were grouped according to PNI quartiles and an identified optimal threshold. Cox regression, restricted cubic spline (RCS) analysis, and subgroup analyses were conducted to evaluate associations between PNI and 30-day and 90-day all-cause mortality.

**Results:**

Among 1,457 critically ill patients with VAP, the all-cause mortality rates were 23.68% at 30 days and 34.32% at 90 days. Patients in the highest PNI quartile exhibited significantly reduced mortality risks compared with the lowest quartile, with an adjusted HR of 0.60 (95% CI, 0.44–0.81) for 30-day mortality and 0.64 (95% CI: 0.50–0.82) for 90-day mortality. RCS analysis revealed a significant non-linear “L”-shaped relationship between PNI and mortality (*p* < 0.001). Below the threshold, patients with higher PNI had significantly lower risk of 30-day mortality (HR = 0.93, 95% CI: 0.91–0.95) and 90-day mortality (HR = 0.94, 95% CI: 0.92–0.96).

**Conclusion:**

A higher PNI at ICU admission was independently associated with lower short-term and long-term mortality in critically ill VAP patients. Routine assessment of PNI could enable early identification of high-risk patients and guide targeted nutritional and immunological interventions.

## 1 Introduction

Ventilator-associated pneumonia (VAP) is a significant and prevalent hospital-acquired infection affecting critically ill patients requiring mechanical ventilation. The incidence of VAP varies widely depending on the clinical setting and patient population, ranging from 5 to 40% in general ICU settings. Specifically, studies have reported an incidence of 9.2% in Portuguese ICUs and 7.48% in tertiary care hospitals (1–3). An epidemiological investigation revealed that the prevalence of ventilator-associated bacterial pneumonia (VABP) was up to 47.9% (4). VAP significantly increased the length of ICU and hospital stays, durations of mechanical ventilation, leading to higher healthcare costs and resource utilization (1, 2). Moreover, VAP remains a major concern in ICUs due to its high mortality rates, which range from 24 to 76% (5, 6). A study in Portugal reported all-cause mortality rates of 24.9% at 28 days, 34.0% at 90 days, and 40.6% at 365 days for VAP patients (2). The presence of drug-resistant pathogens, such as carbapenem-resistant *Acinetobacter baumannii* (CRAB), significantly increased mortality, with a 21-day mortality rate of 25.2% in CRAB-related VAP cases (7). Notably, patients receiving inappropriate empirical antimicrobials exhibited a significantly higher 28-day mortality rate (34.3% vs. 19.5%) (2). Despite advancements in infection control and antimicrobial therapy, accurately assessing mortality risk and identifying high-risk VAP patients remains crucial yet challenging.

Malnutrition and immune dysfunction are highly prevalent in ICU patients and play a crucial role in infection susceptibility, delayed recovery, and poor prognosis. Malnutrition exacerbates the stress response and systemic inflammation, leading to multiorgan failure and other complications (8). In turn, critical illness induced a hypermetabolic and catabolic state, leading to significant nutritional depletion and immune suppression. Given the complex interplay between nutritional status, immune function, and infection-related outcomes, assessing nutritional and immunological status in ICU patients is crucial for risk stratification and early intervention.

The Prognostic Nutritional Index (PNI), calculated from serum albumin levels and lymphocyte counts, serves as an integrated biomarker reflecting both nutritional and immune status. It has been widely studied across various medical conditions, demonstrating its utility in predicting disease outcomes and patient prognosis. Studies have shown that a low PNI score was associated with increased mortality in sepsis patients. For instance, a study in Japan found that, when the PNI dropped below 40, the risk of mortality increased significantly, highlighting its utility in identifying high-risk patients (9). Another study confirmed that a low PNI score was an independent risk factor for all-cause mortality in critically ill patients over various time frames (30, 90, 180, and 365 days) (10). Despite growing evidence linking PNI to adverse outcomes in critically ill patients, its specific prognostic value in VAP remained unclear.

This study aims to evaluate the prognostic significance of the PNI in critically ill patients with VAP by assessing its association with 30-day and 90-day all-cause mortality. Additionally, we seek to explore the potential non-linear relationship between PNI and mortality risk and determine whether a threshold exists for risk stratification, which could guide early risk assessment and personalized clinical interventions.

## 2 Methods

### 2.1 Data source

This study utilized data from the Medical Information Mart for Intensive Care IV (MIMIC-IV, version 3.1), a publicly available critical care database developed by the Massachusetts Institute of Technology (MIT) in collaboration with the Beth Israel Deaconess Medical Center (BIDMC). MIMIC-IV contains de-identified clinical data from patients admitted to BIDMC between 2008 and 2022, ensuring compliance with the Health Insurance Portability and Accountability Act (HIPAA). The dataset integrates structured electronic health records from multiple hospital information systems, including demographic characteristics, admission details, laboratory tests, vital signs, medication administration, organ support interventions, and discharge outcomes. As all data were de-identified, the requirement for individual patient consent was waived in accordance with the Declaration of Helsinki. Access to the database was granted upon the completion of the Collaborative Institutional Training Initiative (CITI) program (Record ID: 64595863).

### 2.2 Patient selection

**Figure 1:**
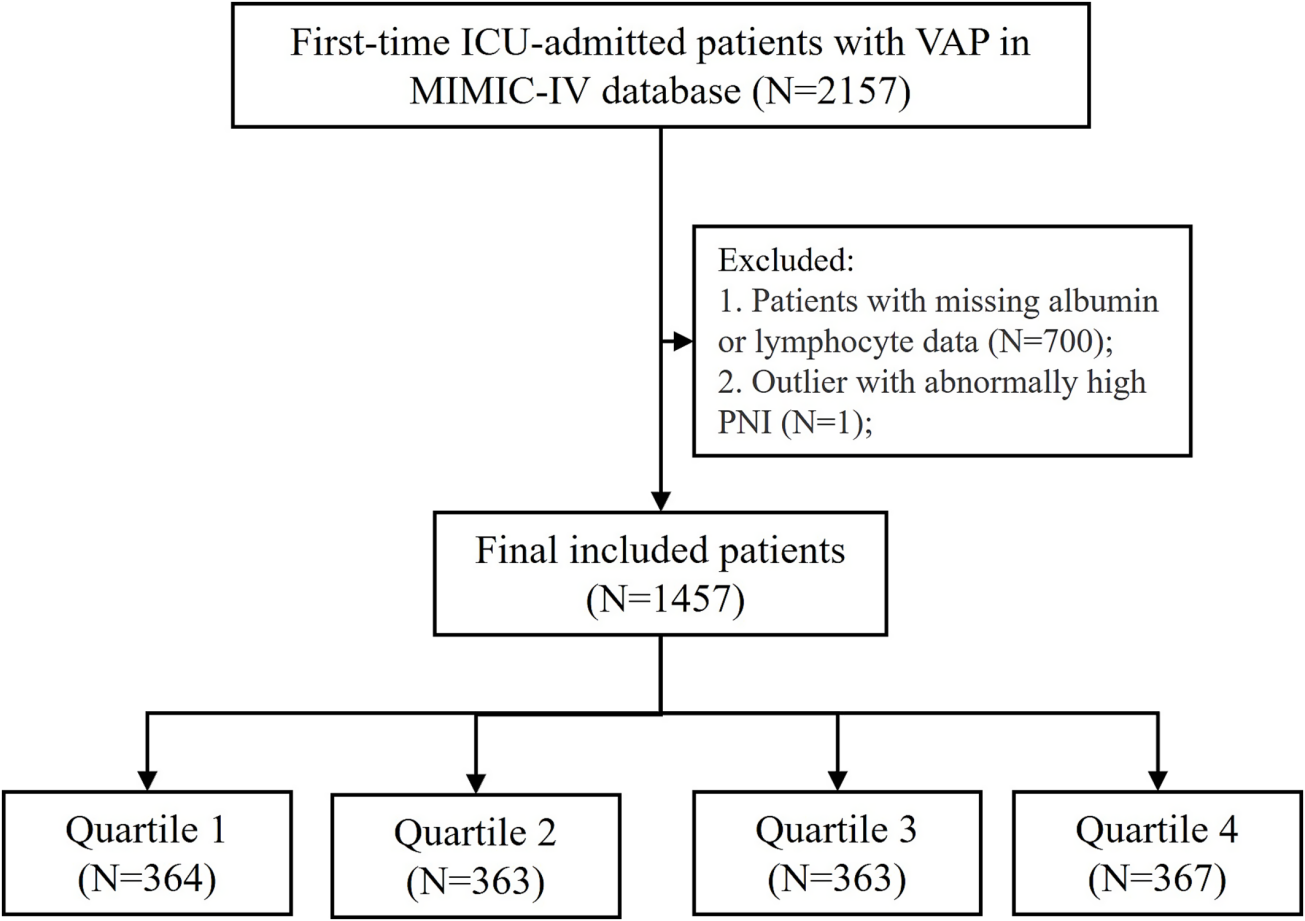
Flowchart of patient inclusion and exclusion criteria for the study. VAP, ventilator-associated pneumonia; PNI, prognostic nutritional index. PNI quartiles: Quartile 1 (≤31.25), Quartile 2 (31.26–36.87), Quartile 3 (36.88–41.65), and Quartile 4 (≥41.66). Cases with PNI > 200 were excluded from the analysis.

Data were extracted from MIMIC IV via structured query language. Patients diagnosed with ventilator-associated pneumonia (VAP) were identified based on ICD-9 and ICD-10 diagnostic codes. The initial cohort included 2,157 patients who were admitted to the ICU for the first time and aged 18 to 100 years. PNI was calculated with data at ICU admission by the formula “PNI = 10 × serum albumin (g/dL) + 0.005 × total lymphocyte counts (/mm^3^)” (11). Cases were excluded if they had missing peripheral blood lymphocyte counts or serum albumin data. Outliers with extreme PNI values were also removed. Finally, a total of 1,457 patients met the final inclusion criteria for analysis and were divided into four groups by PNI quartiles (Figure 1).

### 2.3 Variable extraction

Relevant variables were extracted at ICU admission, encompassing demographic characteristics, vital signs, laboratory measurements, clinical scoring systems, comorbidities, and treatment interventions. Demographic variables included age, sex, and ethnicity. Vital signs included heart rate, systolic blood pressure (SBP), diastolic blood pressure (DBP), mean blood pressure (MBP), respiratory rate, and body temperature. Laboratory parameters covered indicators of hepatic function, renal function, electrolytes, coagulation profiles, and blood gas analysis, including international normalized ratio (INR), prothrombin time (PT), partial thromboplastin time (PTT), alanine aminotransferase (ALT), alkaline phosphatase (ALP), aspartate aminotransferase (AST), total bilirubin, albumin, lactate dehydrogenase (LDH), chloride, anion gap, calcium, potassium, sodium, glucose, blood urea nitrogen (BUN), creatinine, hematocrit, hemoglobin, platelet count, red blood cell count (RBC), red cell distribution width (RDW), white blood cell count (WBC), and differential counts of basophils, eosinophils, lymphocytes, monocytes, and neutrophils. Blood gas variables included oxygen saturation (SpO2), partial pressure of oxygen (PO2), partial pressure of carbon dioxide (PCO2), PaO₂-FiO₂ ratio, arterial pH, and base excess. Clinical severity assessment included the Charlson Comorbidity Index (CCI), Acute Physiology Score III (APS III), Logistic Organ Dysfunction System (LODS), Oxford Acute Severity of Illness Score (OASIS), Sequential Organ Failure Assessment (SOFA), Simplified Acute Physiology Score II (SAPS II), Glasgow Coma Scale (GCS), Systemic Inflammatory Response Syndrome criteria (SIRS), and CURB-65 score. Comorbidities extracted for analysis included congestive heart failure, cerebrovascular disease, chronic pulmonary disease, liver disease, diabetes, renal disease, malignant cancer, acute kidney injury (AKI), and sepsis. Treatment interventions recorded during ICU admission included vasoactive agent use, continuous renal replacement therapy (CRRT), and antibiotic administration.

### 2.4 Grouping and outcomes

Patients were stratified into quartiles based on PNI distribution to explore the baseline difference in clinical outcome and other variables among groups. The primary outcome of this study was 30-day mortality, defined as death occurring within 30 days of ICU admission. The secondary outcome was 90-day mortality, defined as death within 90 days of ICU admission. Survival time was calculated from the date of ICU admission to the date of death or the last follow-up. Patients who remained alive at the study endpoint were considered right-censored at the predefined cutoff date.

### 2.5 Statistical analysis

All statistical analyses were performed using R (version 4.4.2). Survival and categorical variables had no missing values, while continuous variables with more than 25% missingness were excluded, and those with ≤25% missingness were imputed using the random forest method. Collinearity among continuous variables was assessed using Spearman’s correlation matrix. For variables with a correlation coefficient greater than 0.6, the one with the weaker association with the primary outcome was removed to minimize multicollinearity.

Considering the PNI distribution in the dataset (Supplementary Figure 1), PNI values greater than 200 (n = 1) were defined as extreme outliers to keep as many cases as possible. Additionally, sensitivity analyses were conducted by identifying and removing outliers using the conventional 3 × IQR (interquartile range) rule (values lying outside [Q1–3 × IQR, Q3 + 3 × IQR]).

Variable distributions were examined using the Kolmogorov–Smirnov test for normality. Continuous variables were compared between groups using the independent *t*-test or Mann–Whitney U-test, depending on normality, while categorical variables were analyzed using the chi-square test or Fisher’s exact test, as appropriate. Survival analysis was conducted using the Kaplan–Meier method with log-rank tests to compare survival distributions across groups. The association between PNI and mortality was assessed using the Cox proportional hazards regression model. To explore potential non-linear relationships, restricted cubic splines (RCS) were applied to visualize the dose–response association between PNI and the risk of mortality.

## 3 Results

### 3.1 Baseline characteristics of VAP patients grouped by PNI quartiles

A total of 1,457 patients with VAP were included in this study. The median age was 64.06 years, and 64.24% were male. The overall 30-day and 90-day mortality rates were 23.68 and 34.32%, respectively. Patients were stratified into quartiles based on PNI values: Quartile 1 (≤ 31.25), Quartile 2 (31.26–36.87), Quartile 3 (36.88–41.65), and Quartile 4 (≥ 41.66). As shown in Supplementary Table S1, PNI was significantly inversely associated with age, with patients in the lowest quartile (Quartile 1) being the oldest. Patients with the lowest PNI exhibited lower weight, blood pressure, body temperature, PTT, ALT, AST, LDH, albumin, anion gap, calcium, potassium, sodium, blood glucose, hematocrit, hemoglobin, platelets, RBC, RDW, WBC, basophils, eosinophils, monocytes, lymphocytes, neutrophils, PO2, PCO2, PaO_2_-FiO_2_ ratio, and pH (all *p* < 0.05). Additionally, those patients also showed higher CCI, APS III, OASIS, SAPS II, and CURB-65 scores, and more use of vasoactive agents. The prevalence of cerebrovascular disease was the lowest, and that of malignant cancer was the highest in patients with the lowest PNI. Other comorbidities had no significant difference among the PNI quartiles. Meanwhile, patients in the lowest PNI group stayed for a shorter time in the ICU and hospital and exhibited the highest hospital, 30-day, and 90-day mortality.

### 3.2 Survival analysis of PNI and mortality in VAP patients

**Figure 2:**
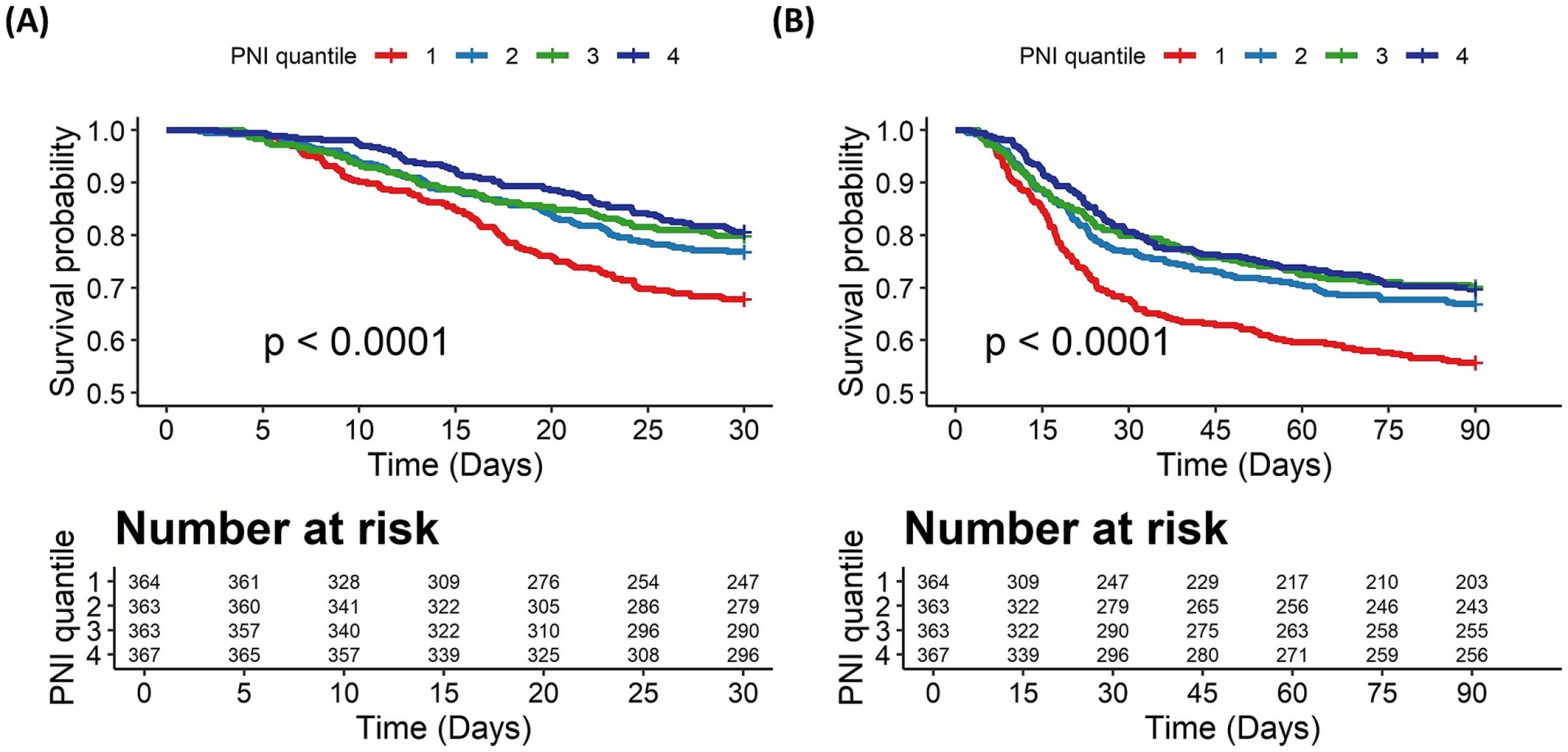
The Kaplan–Meier survival curves for all-cause mortality stratified by PNI quartiles. **(A)** 30-day mortality. **(B)** 90-day mortality. PNI quartiles: Quartile 1 (≤31.25), Quartile 2 (31.26–36.87), Quartile 3 (36.88–41.65), and Quartile 4 (≥41.66). Cases with PNI > 200 were excluded from the analysis.

Kaplan–Meier survival curves were generated to assess survival probability across PNI quartiles (Figure 2). Patients in the lowest PNI quartile (1) exhibited the steepest decline in survival probability, while those in the highest quartile (4) demonstrated the most favorable survival trajectory in the 30-day outcome (log-rank *p* < 0.001, Figure 2A). A similar trend was observed in the 90-day outcome (log-rank p < 0.001, Figure 2B). This divergence in survival probabilities persisted, indicating that PNI is a significant predictor of both short-term and long-term mortality.

Cox proportional hazards models were used to evaluate the independent association between PNI and mortality, adjusting for potential confounders, as shown in Table 1. As a continuous variable, higher PNI was associated with a lower risk of both 30-day (HR = 0.98, 95% CI: 0.97–1.00, *p* = 0.01) and 90-day mortality (HR = 0.98, 95% CI: 0.97–0.99, *p* = 0.002) in the unadjusted model (Model 1). This protective effect remained significant after adjusting for baseline comorbidities and interventions (Model 2) and further adjusting for laboratory parameters and disease severity scores (Model 3), although the HR estimates had attenuated slightly. As a categorical variable (PNI quartiles), a significant inverse trend was observed, with mortality risk progressively decreasing from Quartile 1 (reference) to Quartile 4 across all models in unadjusted and adjusted models, both in 30-day and 90-day mortality. Sensitivity analyses using the 3 × IQR rule to exclude outliers yielded results consistent with the primary analysis, confirming the robustness of the association between PNI and all-cause mortality (Supplementary Table S2).

**Table 1:** Cox regression of PNI and 30-day and 90-day mortality in VAP patients.

PNI	Model 1			Model 2			Model 3		
	HR (95% CI)	*p*-value	P for trend	HR (95% CI)	*p*-value	P for trend	HR (95% CI)	*p*-value	P for trend
30-day mortality									
Continuous	0.98 (0.97–1.00)	0.01		0.99 (0.98–1.00)	0.017		0.99 (0.98–1.00)	0.03	
Q1 (Reference)			<0.001			<0.001			0.002
Q2	0.68 (0.51–0.9)	0.006		0.66 (0.5–0.88)	0.004		0.68 (0.51–0.91)	0.008	
Q3	0.58 (0.44–0.78)	<0.001		0.67 (0.5–0.91)	0.009		0.71 (0.52–0.96)	0.027	
Q4	0.54 (0.40–0.73)	<0.001		0.59 (0.43–0.79)	<0.001		0.6 (0.44–0.81)	0.001	
90-day mortality									
Continuous	0.98 (0.97–0.99)	0.002		0.99 (0.98–1)	0.003		0.99 (0.98–1)	0.005	
Q1 (Reference)			<0.001			<0.001			0.001
Q2	0.68 (0.54–0.86)	0.002		0.67 (0.53–0.85)	0.001		0.66 (0.51–0.84)	<0.001	
Q3	0.6 (0.47–0.77)	<0.001		0.69 (0.54–0.89)	0.004		0.7 (0.54–0.9)	0.005	
Q4	0.6 (0.47–0.76)	<0.001		0.64 (0.5–0.83)	<0.001		0.64 (0.5–0.82)	<0.001	

Model 1, unadjusted. Model 2, covariates including age, congestive heart failure, cerebrovascular disease, chronic pulmonary disease, liver disease, renal disease, malignant cancer, and CRRT, were adjusted. Model 3, covariates including age, congestive heart failure, cerebrovascular disease, chronic pulmonary disease, liver disease, renal disease, malignant cancer, CRRT, INR, PT, PTT, BUN, creatinine, glucose, sodium, chloride, AST, LDH, total bilirubin, SOFA, CCI, and CURB-65, were adjusted. PNI quartiles: Quartile 1 (≤31.25), Quartile 2 (31.26–36.87), Quartile 3 (36.88–41.65), and Quartile 4 (≥41.66). Cases with PNI >200 were excluded from the analysis.

### 3.3 Non-linear relationship between PNI and mortality risk

**Figure 3:**
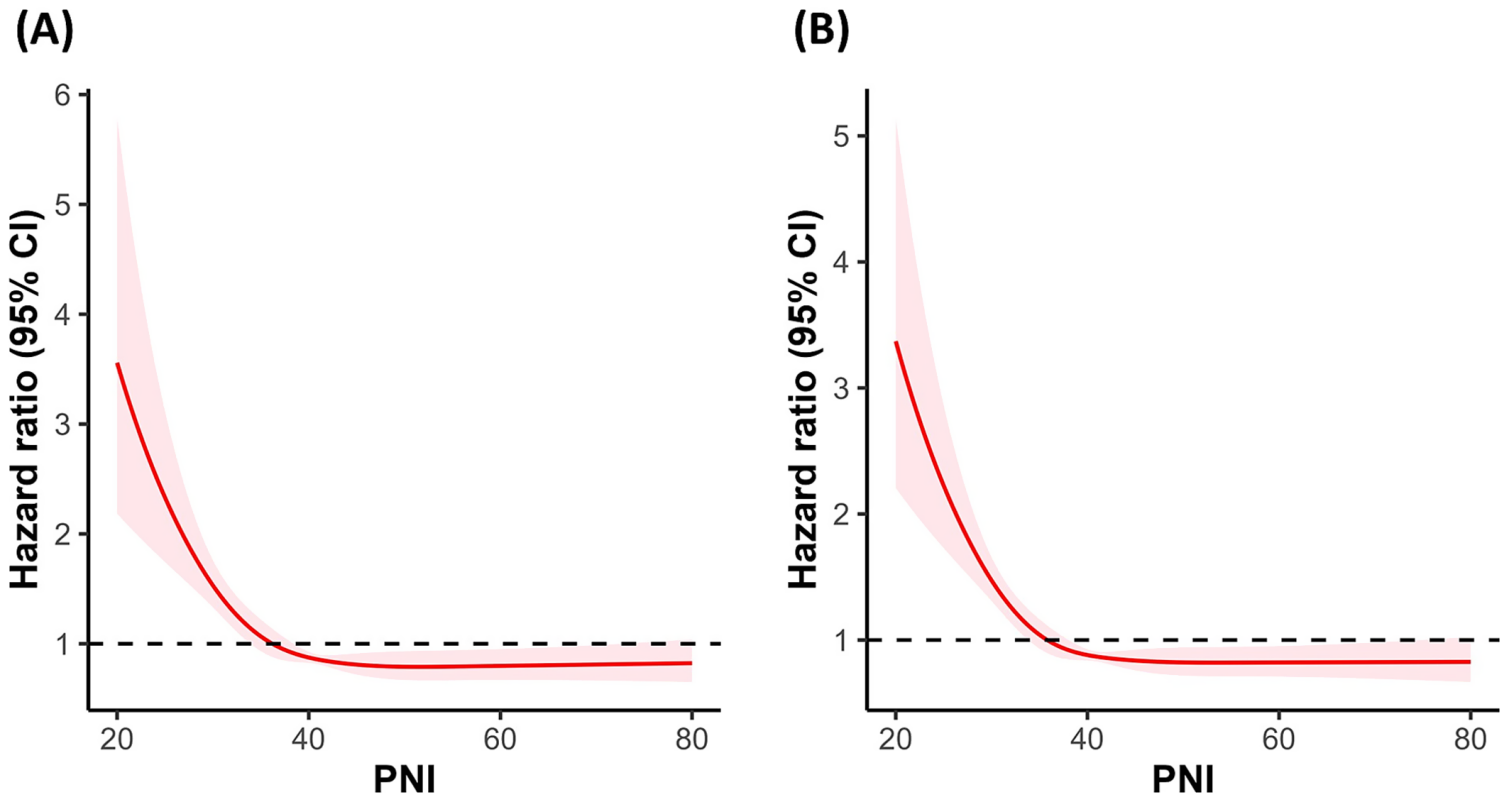
Correlation between the PNI and the hazard ratio for all-cause mortality in VAP patients. **(A)** RCS curve for 30-day mortality. **(B)** RCS curve for 90-day mortality. PNI quartiles: Quartile 1 (≤31.25), Quartile 2 (31.26–36.87), Quartile 3 (36.88–41.65), and Quartile 4 (≥41.66). Cases with PNI > 200 were excluded from the analysis.

To investigate the potential non-linear association between PNI and mortality risk, RCS regression was performed. Figure 3 illustrates the RCS plots for 30-day (Figure 3A) and 90-day (Figure 3B) mortality. The results indicated a significant non-linear association between PNI and mortality risk. Along with PNI increasing, the hazard ratio exhibited an “L”-shaped change. To further explore the potential threshold of PNI, segmented Cox regression analysis was performed, confirming the existence of a threshold effect (Table 2). The potential threshold was identified at PNI = 38.90 for 30-day mortality and PNI = 38.75 for 90-day mortality. The risk decreased by 0.7 and 0.6% when the PNI below threshold gained a one-unit increase in 30-day and 90-day mortality, respectively. For PNI values below the threshold, each one-unit increase in PNI was associated with a 7% and 6% reduction in the risk of 30-day and 90-day mortality, respectively. In contrast, for PNI values above the threshold, no significant association with 30-day or 90-day mortality was observed.

**Table 2:** Threshold effect analysis of PNI on 30-day and 90-day mortality in patients with VAP.

Model	HR (95% CI), *p*-value	
	30-day mortality	90-day mortality
Linear (continuous)	0.98 (0.97, 1), 0.01	0.98 (0.97, 0.99), 0.002
Inflection point (threshold)	38.90	38.75
Two-stage model (< threshold)	0.93 (0.91, 0.95), <0.001	0.94 (0.92, 0.96), <0.001
Two-stage model (≥ threshold)	1 (0.99, 1.01), 0.793	1 (0.99, 1.01), 0.977
Log-likelihood ratio test (*p*-value)	<0.001	<0.001

### 3.4 Subgroup analysis

**Figure 4:**
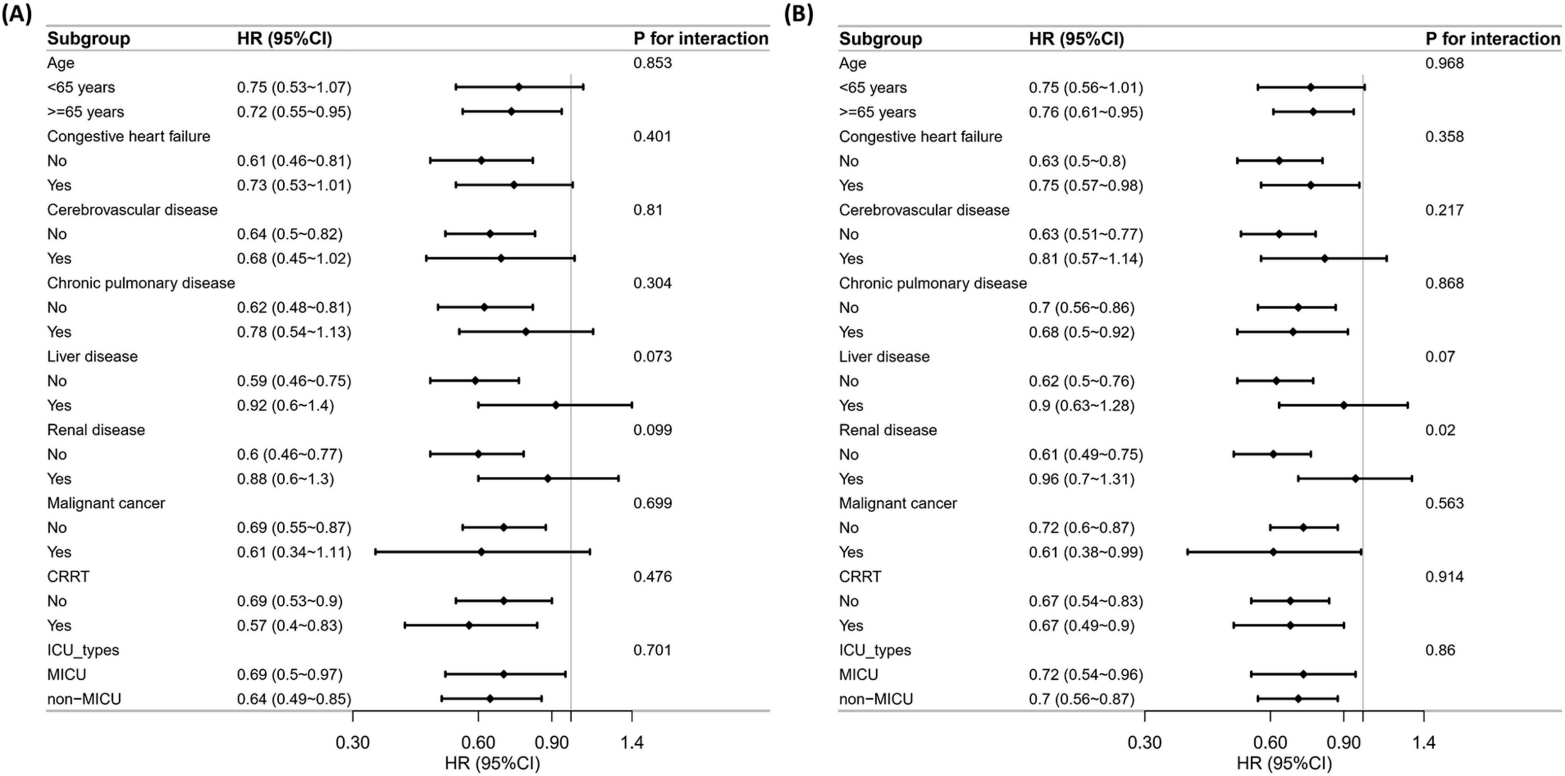
Subgroup analysis of the correlation between PNI and all-cause mortality. **(A)** 30-day mortality. **(B)** 90-day mortality.

Subgroup analyses were performed on age, congestive heart failure, cerebrovascular disease, chronic pulmonary disease, liver disease, renal disease, malignant cancer, CRRT, and ICU types to assess the robustness of the association between low PNI and mortality risk in critically ill VAP patients. According to the distribution of ICU types in our study cohort (Supplementary Figure 2), we categorized patients into the MICU and non-MICU groups, as the numbers in other ICU types were too small. For 30-day mortality (Figure 4A), no significant interactions were found across subgroups (P for interaction > 0.05). For 90-day mortality (Figure 4B), a significant interaction was observed in the renal disease subgroup (P for interaction = 0.020). Low PNI was associated with a higher mortality risk in patients without renal disease (HR = 0.61), while no significant effect was found in those with renal disease (HR = 0.96).

### 3.5 Comparative predictive performance of PNI and established clinical scores

**Figure 5:**
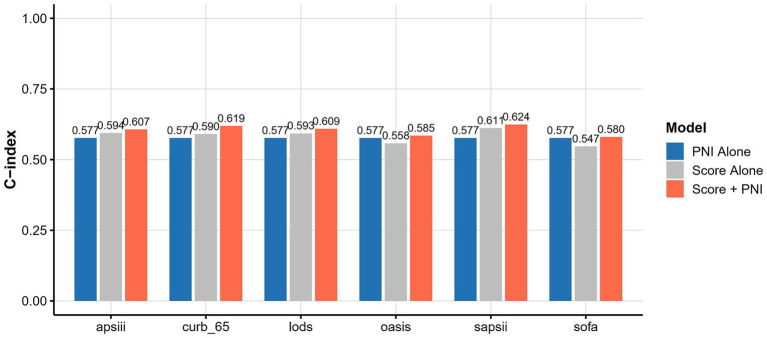
C-index comparison of PNI, clinical scores, and combined models for 30-day mortality prediction.

We further compared the predictive performance of PNI (both as a continuous variable and as a quartile-based categorical variable) with established clinical scoring systems for 30-day and 90-day mortality. As shown in Figure 5, the C-index of PNI alone for predicting 30-day mortality was 0.577, which was higher than OASIS (0.558) and SOFA (0.547), but slightly lower than CURB-65 (0.590) and SAPS II (0.611). Notably, the addition of PNI to each clinical score consistently improved model discrimination, with the combined models achieving higher C-indices compared to either PNI or the clinical scores alone. A similar trend was observed when PNI was analyzed as a four-level categorical variable (quartiles), as illustrated in Supplementary Figure 3. For 90-day mortality, the predictive performance of PNI, PNI quartile, and clinical scores displayed the same pattern, as shown in Supplementary Figures 4, 5, respectively.

## 4 Discussion

This study demonstrated a significant inverse association between PNI and all-cause mortality in critically ill patients with VAP. Patients in the highest PNI quartile exhibited a 40% lower risk of mortality compared to those in the lowest quartile at 30 days (HR: 0.60, 95% CI: 0.44–0.81, *p* = 0.001; P for trend = 0.002) and 36% lower risk of mortality at 90 days (HR: 0.64, 95% CI: 0.5–0.82, *p* < 0.001; P for trend = 0.001) after full adjustment for confounders. Moreover, RCS analysis confirmed a significant non-linear “L”-shaped relationship between PNI and mortality, revealing a clear threshold effect at a PNI value of approximately 38.90 at 30-day mortality and 38.75 at 90-day mortality. When PNI was below the threshold, patients with lower PNI showed significantly poorer survival outcomes at both 30 days and 90 days. PNI, calculated from serum albumin levels and peripheral lymphocyte counts, is a composite biomarker that effectively integrates nutritional and immune statuses, both of which are critical to patient outcomes in intensive care settings. Critically ill patients frequently exhibited simultaneous nutritional depletion and immune suppression, which often synergistically exacerbated disease severity, complicating clinical management and adversely impacting patient prognosis (12, 13). Serum albumin plays pivotal physiological roles, including maintaining plasma colloid osmotic pressure, exerting antioxidant and anticoagulant effects, regulating immune responses, and preserving vascular endothelial integrity. In critically ill patients, hypoalbuminemia is common, primarily attributed to inadequate nutritional intake, increased protein catabolism driven by systemic inflammation, impaired hepatic protein synthesis, and enhanced capillary permeability leading to protein leakage into interstitial spaces (14). Prior studies have consistently demonstrated a robust association between hypoalbuminemia and adverse outcomes in severe infections (15–17), including prolonged duration of mechanical ventilation and elevated short-term and long-term mortality rates. Lymphocytes constitute a fundamental component of cellular immunity, and lymphopenia serves as an important indicator of compromised immune defense. Lymphocytopenia was commonly observed in sepsis and was associated with increased mortality. This condition reflected an immunosuppressive state, where the body’s ability to fight infections was compromised. Studies have shown that low lymphocyte counts and proportional shifts within lymphoid cell populations were linked to poor outcomes, indicating a weakened immune response (18–20).

Previous studies have widely investigated the prognostic value of the PNI across various medical conditions, including chronic diseases, surgical and oncological contexts, and acute and critical conditions. For instance, Liu et al. found that higher PNI levels were associated with lower diabetes-related and cardiovascular mortality in patients with uncontrolled diabetes (21). Low PNI was reported to be inversely related to both all-cause and cardiovascular mortality among hypertensive patients (22). In the previous study, PNI was used to predicting mortality of AECOPD patients. And in that study, PNI displayed lower AUROC than SAPS II (23). Our previous studies found that PNI was a significant predictor of 30-day and 90-day mortality in community-acquired pneumonia (CAP) patients (24). It outperformed traditional scoring systems such as CURB-65, suggesting its potential as a superior prognostic tool in acute infections. VAP represented a distinct clinical entity characterized by a high degree of severity, rapid disease progression, significant inflammatory responses, and notably high morbidity and mortality. Critically ill patients who developed VAP frequently exhibited severe physiological derangements, pronounced immunosuppression, and heightened metabolic stress, all of which profoundly influence prognosis and complicate management strategies. Compared to CAP, VAP was associated with higher severity and mortality rates (25). Therefore, we further explored the specific prognostic value of PNI in VAP.

The accurate prediction of mortality in VAP patients remains a critical clinical challenge, prompting the development of various predictive indices and models. Monocyte chemoattractant protein-1 (MCP-1), RDW, and serum procalcitonin level have been reported to be promising biomarkers of mortality in patients with VAP. MCP-1 also performed well in recognizing early-stage VAP (26–28). Comorbidities and severity scores were also used for assessing the mortality of VAP, including Acute Physiology and Chronic Health Evaluation II (APACHE II), Clinical Pulmonary Infection Score (CPIS), Sequential Organ Failure Assessment (SOFA), Charlson Comorbidity Index score, and PIRO, predisposition, insult, response, organ dysfunction. The elements of the PIRO concept are as follows: predisposition (chronic illness, age, and comorbidities), insult (injury, bacteremia, and endotoxin), response (neutropenia, hypoxemia, and hypotension), and organ dysfunction (29–32). The presence of multidrug-resistant organisms, such as carbapenem-resistant bacterial pathogens, *Acinetobacter baumanii* and *Candida*, may predict poor outcomes of VAP (30, 33). However, existing biomarkers or predictive tools predominantly rely on clinical and microbiological data without adequately addressing the underlying nutritional and immunological status—critical factors known to influence patient outcomes significantly. To our knowledge, this is the first study to specifically evaluate the PNI, a composite biomarker reflecting both nutritional reserves and immune competence, in critically ill patients with VAP. Given the intrinsic complexity, severe clinical presentation, and poor prognosis of patients with VAP, incorporating PNI into mortality risk stratification frameworks represented an important innovation. Thus, our findings bridge a significant gap in the current literature, highlighting the potential utility of nutritional and immunological assessments in refining mortality predictions and facilitating targeted, individualized clinical interventions in VAP management.

In our subgroup analysis, renal disease significantly modified the predictive value of PNI for 90-day mortality but did not affect its predictive ability for 30-day mortality. Specifically, low PNI was strongly associated with increased 90-day mortality in patients without renal disease (HR = 0.61), whereas no significant relationship was observed among patients with renal disease (HR = 0.96). This discrepancy may reflect the underlying differences in pathophysiological mechanisms related to nutritional and immune status between patients with and without chronic renal impairment. Patients with chronic renal disease often exhibit a persistently altered nutritional and inflammatory status, characterized by chronic hypoalbuminemia, sustained inflammation, and lymphocyte dysfunction (34–36). In these patients, long-standing malnutrition and immune dysregulation could diminish the prognostic sensitivity of acute changes in PNI, thereby attenuating its ability to distinguish differences in long-term outcomes. Conversely, patients without pre-existing renal dysfunction were more likely to possess relatively intact baseline nutritional and immune reserves. Consequently, an acute decline in nutritional and immunological status (as reflected by the low PNI) more sensitively identified patients who were vulnerable to poor long-term outcomes. The absence of such interaction at 30 days can be explained by the nature of early mortality predictors. Although chronic conditions such as renal disease profoundly influence long-term outcomes, early mortality was predominantly influenced by acute exacerbations in patients’ physiological, nutritional, and immunological status caused by VAP. Given that PNI effectively captured acute nutritional and immune impairments, it remained predictive of short-term mortality across various subgroups, regardless of underlying chronic comorbidities. Therefore, at 30 days, the acute deterioration in nutritional and immunological status reflected in the low PNI was a consistently significant risk factor for mortality, overshadowing the moderating effects of chronic renal disease.

Furthermore, our analysis revealed that receiving CRRT did not influence the predictive ability of PNI for either 30-day or 90-day mortality. This finding likely reflects the fundamentally different clinical roles of chronic renal disease and CRRT treatment. While chronic renal impairment represented a stable and enduring alteration in patients’ nutritional and inflammatory profiles, CRRT served as an acute or subacute therapeutic intervention aimed at rapidly correcting fluid, electrolyte, and acid–base imbalances and removing inflammatory mediators. However, CRRT typically did not substantially alter the underlying nutritional and immunological reserves of patients. Therefore, the nutritional and immune deficits reflected by low PNI remained significant predictors of poor prognosis, irrespective of CRRT application. These results underscored the robustness and clinical relevance of PNI as a marker of baseline nutritional and immune status and its utility in risk stratification across diverse clinical scenarios.

Our study has several important strengths and clinical implications. To our knowledge, this is the first investigation to comprehensively evaluate the prognostic significance of the PNI specifically in critically ill patients diagnosed with VAP. We identified a clear non-linear association between PNI and mortality, further determining a clinically relevant threshold value for patient stratification. This approach not only enhances the prognostic accuracy but also simplifies its application in clinical practice by providing clinicians with a straightforward criterion for risk assessment. From a clinical perspective, the introduction of PNI into routine risk stratification protocols in ICU settings has several practical implications. First, regular and repeated assessment of PNI could facilitate early identification of patients at high risk for poor outcomes and dynamically monitor nutritional and immunological trajectory, enabling timely initiation and adjustment of targeted interventions. Moreover, patients with low PNI scores at admission could benefit from early multidisciplinary interventions.

Immunonutrition refers to the administration of specific nutrients, including *ω*-3 polyunsaturated fatty acids, arginine, glutamine, sulfur-containing amino acids, antioxidants, and nucleotides, alone or in combination, with the goal of modulating inflammation and supporting recovery. The efficacy of immunonutrition has been widely explored in patients with cancer, surgical stress, and trauma, with accumulating evidence also supporting its application in critically ill patients. A multicenter prospective observational study further demonstrated that immunonutrition with high protein, arginine, nucleotides, and polyunsaturated fatty acids during ICU admission was independently associated with higher protein intake, lower requirements for vasopressor and renal replacement therapies, and a trend toward improved 28-day survival (37). Increasing intake of omega-3 fatty acids, arginine, and dietary nucleotides, while maintaining equal protein intake, was also linked to a significantly shorter hospital stay, along with enhanced immune cytokine responses and modulation of lymphocyte subsets (38, 39). Therefore, the targeted use of immunonutrition may represent a pragmatic strategy for improving the prognosis of VAP patients identified as high risk based on low PNI values.

The benefit of immunomodulators in the management of VAP remains uncertain. High-dose intravenous immunoglobulin (IVIG) played an important role in immune dysfunction as an anti-inflammatory and immunomodulatory therapy (40). In addition, it has been investigated as adjunctive therapy in a range of severe infectious and inflammatory lung conditions, including steroid-refractory immune checkpoint inhibitor (ICI)-related pneumonitis and severe community-acquired pneumonia (sCAP). Recent studies have reported that IVIG may help improve oxygenation, facilitate clinical recovery, and reduce mortality in selected patients with dysregulated inflammation or low baseline immunoglobulin levels (41). In the CIGMA trial, the use of trimodulin (a human polyvalent immunoglobulin preparation) was associated with more rapid normalization of inflammatory markers and lower mortality in sCAP patients with low lymphocyte or IgM levels (42). However, acute kidney injury, thromboembolism, and severe hemolysis have been reported, particularly at higher cumulative doses (43, 44). To date, IVIG has not been recommended for routine use in VAP or sepsis, and its administration should be reserved for carefully selected patients after a thorough risk–benefit assessment. While accumulating evidence supported the potential benefits of high-dose IVIG, current data were limited, and its safety and efficacy in this population remained to be firmly established. Therefore, well-designed prospective clinical trials, or targeted trial emulation using high-quality real-world data, are urgently needed to determine the effect and optimal strategies in VAP patients with low PNI. Other immunomodulatory drugs have also garnered interest. Ulinastatin and thymosin alpha 1 were reported to improve mortality and decrease inflammation in patients with sepsis (45).

Emerging research highlights the PI3K/AKT/mTOR pathway as a crucial molecular link between nutrition, metabolism, and immune function. As an intracellular metabolic sensor, mTOR integrates signals from nutrients (amino acids, glucose, and fatty acids) and adipokines such as leptin. Its oscillatory activity precisely regulates the balance between effector T cell activation and regulatory T cell (Treg) proliferation. Notably, both excessive activation and chronic inhibition of mTOR can disrupt immune homeostasis, leading to the abnormal expansion of Tregs and compromised effector cell responses (46, 47). Murine studies have shown that moderate caloric restriction without inducing overt malnutrition suppressed mTOR signaling, drove metabolic reprogramming toward glycolysis and autophagy, and attenuated excessive lymphocyte recruitment and inflammation. This not only limits tissue injury during infection but can also enhance pathogen clearance, as demonstrated in *Mycobacterium tuberculosis*-infected mice (48). However, chronic or severe malnutrition leads to persistent mTOR suppression and elevated Treg dominance, which may inhibit effector lymphocyte proliferation and reduce host defense capacity (46), thereby increasing susceptibility to secondary infections such as VAP in clinical settings. It was reported that oral RTB101, an mTOR inhibitor, increased interferon-induced antiviral gene expression and reduced laboratory-confirmed respiratory tract infections (RTIs) compared to placebo in adults over 65 years (49). However, the use of mTOR inhibitors as immunomodulators in VAP patients with low PNI may not be appropriate, as chronic malnutrition in these patients likely leads to sustained mTOR suppression.

Several limitations of this study warrant consideration. First, the diagnosis of VAP in this study was determined using ICD-9/10 codes, which may be subject to misclassification bias. The accuracy of administrative coding may vary among clinicians and institutions, potentially leading to under- or over-identification of VAP cases. Although the use of standardized codes is a widely accepted approach in large database research, future studies with prospectively adjudicated or microbiologically confirmed VAP cases are warranted to validate our findings. Second, as a retrospective, single-center observational study based on the MIMIC-IV database, inherent limitations, such as potential selection bias and residual confounding, cannot be entirely ruled out, despite our extensive adjustment for relevant covariates. Prospective multicenter studies are needed to confirm the generalizability of these findings across diverse patient populations and clinical settings. Additionally, while we applied multiple imputation to address missing data, this statistical technique cannot fully replace actual recorded values, which may affect the robustness of our results. Finally, our study focused on baseline PNI values at ICU admission without considering dynamic changes during hospitalization; thus, further studies should investigate the prognostic implications of serial PNI measurements. Future research should involve prospective, multicenter cohorts to validate the predictive utility and optimal threshold of PNI. Moreover, integrating PNI with other clinical or biological markers into comprehensive predictive models or artificial intelligence-based systems may enhance prognostic accuracy and guide personalized clinical interventions more effectively.

In conclusion, our study demonstrated that a lower PNI at ICU admission was independently associated with increased 30-day and 90-day all-cause mortality in critically ill patients with VAP. The identified non-linear relationship and clinically relevant threshold value further enhance the clinical utility of PNI in stratifying mortality risk. Incorporating routine PNI assessment into clinical practice could facilitate early identification of high-risk patients, thereby guiding timely nutritional and immunological interventions and ultimately improving patient outcomes.

## Data Availability

Publicly available datasets were analyzed in this study. This data can be found here: https://physionet.org/content/mimiciv/3.1/.
