# Supplementary material for: Association between prognostic nutritional index and all-cause mortality in critically ill patients with ventilator-associated pneumonia: a retrospective study based on MIMIC-IV database
Source: Front Nutr. 2025 Aug 18;12:1605032. doi: 10.3389/fnut.2025.1605032 (PMC12399405; doi:10.3389/fnut.2025.1605032)
Supplement: Supplementary file 1 [file Data_Sheet_1.docx]

supplementary material:


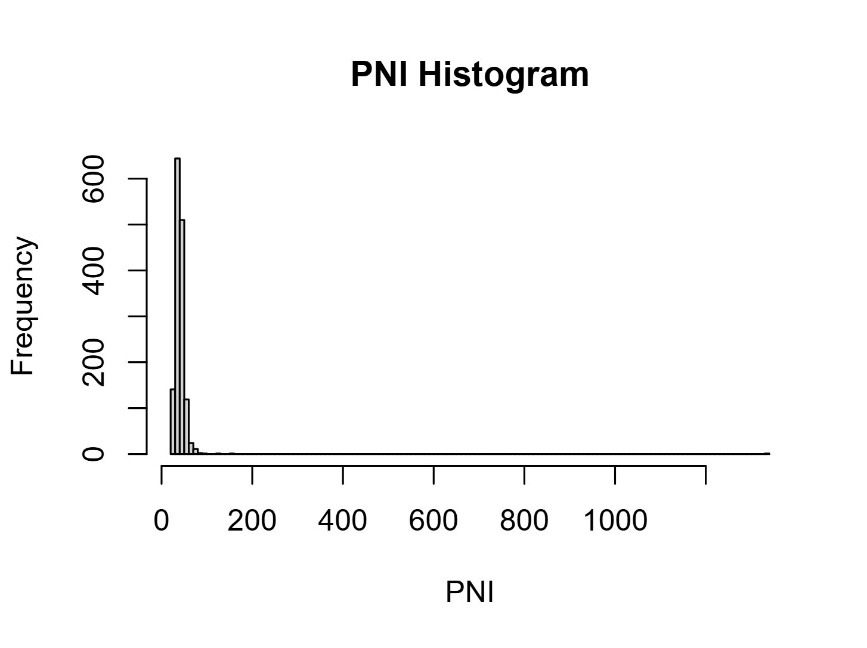


**Supplementary Figure 1** The histogram exhibiting distribution of PNI in the dataset.

**Supplementary Table S1** Baseline characteristics in VAP patients grouped by PNI quartiles.

| Variables | Total  (n = 1457) | Quantile 1  (n = 364) | Quantile 2  (n = 363) | Quantile 3  (n = 363) | Quantile 4  (n = 367) | *P* |
| --- | --- | --- | --- | --- | --- | --- |
|  |  |  |  |  |  |  |
| Age, year | 64.06 (53.10, 73.85) | 67.35 (58.13,75.53) | 66.34 (55.23,76.54) | 62.14 (51.88,71.41) | 59.20 (47.60,70.97) | **<.001** |
| Gender, male | 936 (64.24) | 245 (67.31) | 232 (63.91) | 233 (64.19) | 226 (61.58) | 0.451 |
| Race, n (%) |  |  |  |  |  | 0.443 |
| White | 790 (54.22) | 212 (58.24) | 203 (55.92) | 185 (50.96) | 190 (51.77) |  |
| Black | 175 (12.01) | 35 (9.62) | 46 (12.67) | 46 (12.67) | 48 (13.08) |  |
| Yellow | 40 (2.75) | 12 (3.30) | 11 (3.03) | 10 (2.75) | 7 (1.91) |  |
| Others | 452 (31.02) | 105 (28.85) | 103 (28.37) | 122 (33.61) | 122 (33.24) |  |
| Weight, Kg | 91.50 (76.90, 107.60) | 88.25 (75.50,102.35) | 90.40 (76.75,105.41) | 93.00 (77.46,108.15) | 94.40 (79.18,116.30) | **<.001** |
| Heart Rate, bpm | 134.00 (120.00, 148.00) | 132.50 (119.00,147.00) | 133.00 (121.00,148.00) | 133.00 (119.00,147.00) | 136.00 (123.00,150.00) | 0.061 |
| SBP, mmHg | 181.00 (165.00, 200.00) | 179.00 (161.75,198.00) | 185.00 (167.00,202.00) | 182.00 (165.00,201.50) | 181.00 (165.00,200.00) | **0.035** |
| DBP, mmHg | 116.00 (100.00, 134.00) | 111.50 (97.75,127.00) | 118.00 (100.00,135.50) | 116.00 (101.00,134.00) | 119.00 (102.00,136.00) | **0.005** |
| MBP, mmHg | 142.00 (122.00, 199.00) | 135.00 (118.00,176.25) | 144.00 (120.00,193.50) | 141.00 (123.00,200.50) | 150.00 (127.00,220.00) | **<.001** |
| Respiratory Rate, bpm | 40.50 (35.00, 47.00) | 40.00 (35.00,45.00) | 40.00 (36.00,46.00) | 41.00 (35.00,48.00) | 41.00 (36.00,47.50) | **0.022** |
| Temperature, ℃ | 39.00 (38.33, 39.44) | 38.83 (38.17,39.33) | 38.89 (38.31,39.44) | 39.06 (38.39,39.50) | 39.22 (38.56,39.50) | **<.001** |
| PNI | 39.25 (34.31, 44.85) | 31.25 (28.32,33.00) | 36.87 (35.54,38.05) | 41.65 (40.52,43.05) | 49.31 (46.55,54.12) | **<.001** |
| INR | 1.50 (1.30, 2.20) | 1.50 (1.30,2.00) | 1.60 (1.30,2.30) | 1.50 (1.30,2.20) | 1.60 (1.40,2.50) | **0.036** |
| PT, s | 17.00 (14.50, 23.70) | 16.75 (14.47,21.50) | 17.10 (14.50,25.10) | 16.70 (14.15,23.55) | 17.60 (14.70,26.30) | **0.023** |
| PTT, s | 57.70 (35.50, 129.00) | 47.20 (35.40,109.65) | 53.90 (34.70,132.75) | 59.30 (35.75,126.05) | 70.50 (37.35,141.00) | **0.033** |
| ALT, U/L | 67.00 (32.00, 174.00) | 49.00 (25.00,116.50) | 71.00 (33.00,199.70) | 70.00 (34.00,160.50) | 84.00 (39.00,194.50) | **<.001** |
| ALP, U/L | 141.00 (90.00, 233.00) | 140.00 (89.75,234.25) | 144.00 (95.00,226.00) | 132.00 (88.00,221.00) | 143.00 (89.00,256.50) | 0.703 |
| AST, U/L | 99.61 (46.00, 247.00) | 82.00 (40.00,168.25) | 102.00 (49.00,261.00) | 99.00 (48.50,243.50) | 116.00 (53.00,328.00) | **<.001** |
| Total Bilirubin, mg/dL | 1.00 (0.60, 2.30) | 1.00 (0.50,2.20) | 1.00 (0.50,2.40) | 1.00 (0.60,1.95) | 1.10 (0.60,3.10) | **0.013** |
| Albumin, g/dL | 3.10 (2.70, 3.50) | 2.60 (2.30,2.80) | 3.00 (2.80,3.20) | 3.30 (3.10,3.60) | 3.60 (3.20,4.00) | **<.001** |
| LDH, U/L | 451.00 (308.00, 697.00) | 427.50 (289.75,627.75) | 438.00 (313.00,695.50) | 446.96 (302.50,687.03) | 490.00 (330.59,815.00) | **0.002** |
| Chloride, mmol/L | 111.00 (107.00, 116.00) | 111.00 (107.00,116.00) | 112.00 (107.50,116.00) | 111.00 (107.00,115.00) | 111.00 (107.00,116.00) | 0.140 |
| Anion gap, mmol/L | 19.00 (16.00, 23.00) | 18.00 (15.00,22.00) | 19.00 (16.00,23.00) | 19.00 (16.00,23.00) | 20.00 (17.00,24.00) | **<.001** |
| Calcium, mg/dL | 9.30 (8.80, 10.00) | 8.90 (8.50,9.50) | 9.30 (8.80,9.80) | 9.50 (9.00,9.90) | 9.80 (9.20,10.40) | **<.001** |
| Potassium, mmol/L | 5.20 (4.70, 5.80) | 5.00 (4.60,5.60) | 5.20 (4.80,5.80) | 5.20 (4.60,5.80) | 5.20 (4.70,5.90) | **0.006** |
| Sodium, mmol/L | 148.00 (143.00, 151.00) | 146.00 (142.00,150.00) | 148.00 (144.00,151.50) | 148.00 (143.50,151.00) | 148.00 (144.00,153.00) | **<.001** |
| Glucose, mg/dL | 225.00 (176.00, 317.00) | 212.00 (164.00,290.50) | 229.00 (181.00,332.50) | 224.00 (176.50,318.00) | 241.00 (183.50,321.00) | **<.001** |
| BUN, mg/dL | 54.00 (30.00, 89.00) | 53.50 (29.75,88.00) | 56.00 (33.00,94.50) | 48.00 (28.00,82.50) | 58.00 (32.00,89.50) | **0.023** |
| Creatinine, mg/dL | 1.80 (1.10, 3.90) | 1.90 (1.00,3.73) | 1.90 (1.10,4.30) | 1.70 (1.00,3.30) | 1.90 (1.20,4.00) | 0.158 |
| Hematocrit, % | 35.00 (31.60, 39.70) | 33.10 (29.70,36.92) | 35.00 (31.90,39.55) | 35.60 (32.50,40.00) | 36.90 (32.80,41.75) | **<.001** |
| Hemoglobin, g/dL | 11.40 (10.20, 12.90) | 10.70 (9.50,12.20) | 11.30 (10.20,12.90) | 11.60 (10.45,13.05) | 12.00 (10.60,13.60) | **<.001** |
| Platelet, K/uL | 352.00 (241.00, 500.00) | 326.00 (198.75,430.50) | 340.00 (231.00,493.50) | 378.00 (258.50,518.00) | 382.00 (266.50,524.50) | **<.001** |
| RBC, m/uL | 3.83 (3.39, 4.36) | 3.58 (3.25,4.04) | 3.82 (3.44,4.30) | 3.94 (3.48,4.38) | 4.02 (3.52,4.61) | **<.001** |
| RDW, % | 17.00 (15.40, 19.40) | 16.80 (15.50,18.90) | 16.90 (15.30,19.50) | 16.80 (15.20,19.00) | 17.40 (15.50,20.50) | **0.044** |
| WBC, K/uL | 20.00 (15.10, 27.00) | 18.05 (13.57,24.92) | 19.50 (14.30,25.35) | 20.30 (15.45,26.10) | 23.80 (18.45,32.65) | **<.001** |
| Basophils, K/uL | 0.05 (0.02, 0.10) | 0.03 (0.01,0.05) | 0.05 (0.02,0.08) | 0.05 (0.03,0.09) | 0.07 (0.04,0.16) | **<.001** |
| Eosinophils, K/uL | 0.23 (0.08, 0.46) | 0.15 (0.03,0.30) | 0.20 (0.08,0.40) | 0.26 (0.10,0.47) | 0.35 (0.15,0.66) | **<.001** |
| Lymphocytes, K/uL | 1.48 (0.98, 2.18) | 0.97 (0.63,1.31) | 1.37 (0.93,1.69) | 1.68 (1.22,2.16) | 2.69 (1.94,3.75) | **<.001** |
| Monocytes, K/uL | 1.02 (0.66, 1.56) | 0.77 (0.47,1.12) | 0.97 (0.64,1.36) | 1.05 (0.75,1.59) | 1.39 (0.94,2.21) | **<.001** |
| Neutrophils, K/uL | 13.71 (9.38, 19.44) | 11.72 (8.32,17.52) | 13.07 (8.64,18.04) | 13.77 (9.48,18.95) | 16.09 (11.42,23.19) | **<.001** |
| SpO2, % | 100.00 (100.00, 100.00) | 100.00 (100.00,100.00) | 100.00 (100.00,100.00) | 100.00 (100.00,100.00) | 100.00 (100.00,100.00) | **0.002** |
| PO2, mmHg | 209.00 (157.00, 305.00) | 199.31 (144.00,290.75) | 203.00 (159.00,303.00) | 208.00 (157.00,295.50) | 230.00 (168.50,327.00) | **0.001** |
| PCO2, mmHg | 57.00 (49.00, 69.00) | 56.00 (47.75,68.00) | 58.00 (50.00,68.00) | 56.37 (48.82,69.00) | 59.00 (49.00,72.00) | 0.062 |
| PaO2-FiO2 ratio, mmHg | 393.00 (298.18, 512.50) | 365.00 (270.00,481.50) | 390.00 (309.29,510.42) | 405.71 (288.75,507.62) | 413.75 (321.25,551.96) | **<.001** |
| pH | 7.50 (7.46, 7.53) | 7.48 (7.44,7.52) | 7.49 (7.45,7.53) | 7.50 (7.47,7.53) | 7.50 (7.47,7.54) | **<.001** |
| Base excess, mmol/L | 6.00 (3.00, 10.00) | 6.00 (2.00,9.00) | 6.00 (3.00,10.00) | 7.00 (4.00,10.00) | 7.00 (4.00,11.00) | **<.001** |
| CCI | 5.00 (3.00, 7.00) | 5.00 (3.00,7.00) | 5.00 (3.00,7.00) | 4.00 (2.00,7.00) | 4.00 (3.00,7.00) | **<.001** |
| APS III | 55.00 (42.00, 72.00) | 58.00 (45.00,75.25) | 55.00 (43.50,72.00) | 52.00 (39.50,65.00) | 55.00 (40.00,73.00) | **<.001** |
| LODS | 7.00 (5.00, 9.00) | 7.00 (5.00,9.00) | 7.00 (5.00,9.00) | 7.00 (5.00,9.00) | 7.00 (5.00,9.00) | **0.002** |
| OASIS | 38.00 (33.00, 43.00) | 39.00 (33.00,44.00) | 38.00 (33.00,43.00) | 37.00 (32.00,42.00) | 37.00 (32.00,43.00) | **0.003** |
| SOFA | 7.00 (4.00, 9.00) | 7.00 (5.00,10.00) | 7.00 (4.00,9.00) | 6.00 (4.00,9.00) | 7.00 (4.00,10.00) | **0.007** |
| SAPS II | 43.00 (33.00, 53.00) | 45.00 (36.00,56.00) | 44.00 (34.00,53.00) | 41.00 (32.00,48.00) | 42.00 (31.00,54.00) | **<.001** |
| GCS | 15.00 (15.00, 15.00) | 15.00 (15.00,15.00) | 15.00 (15.00,15.00) | 15.00 (15.00,15.00) | 15.00 (15.00,15.00) | 0.160 |
| SIRS | 3.00 (2.00, 4.00) | 3.00 (2.00,4.00) | 3.00 (2.00,4.00) | 3.00 (2.00,3.00) | 3.00 (2.00,4.00) | 0.065 |
| CURB-65 | 2.00 (2.00, 3.00) | 3.00 (2.00,3.00) | 2.00 (2.00,3.00) | 2.00 (2.00,3.00) | 2.00 (2.00,3.00) | **<.001** |
| Congestive Heart Failure, n (%) | 497 (34.11) | 110 (30.22) | 137 (37.74) | 123 (33.88) | 127 (34.60) | 0.201 |
| Cerebrovascular Disease, n (%) | 321 (22.03) | 64 (17.58) | 73 (20.11) | 91 (25.07) | 93 (25.34) | **0.026** |
| Chronic Pulmonary Disease, n (%) | 408 (28.00) | 110 (30.22) | 108 (29.75) | 94 (25.90) | 96 (26.16) | 0.414 |
| Liver Disease, n (%) | 290 (19.90) | 64 (17.58) | 66 (18.18) | 70 (19.28) | 90 (24.52) | 0.075 |
| Diabetes, n (%) | 490 (33.63) | 122 (33.52) | 134 (36.91) | 115 (31.68) | 119 (32.43) | 0.455 |
| Renal Disease, n (%) | 359 (24.64) | 97 (26.65) | 99 (27.27) | 80 (22.04) | 83 (22.62) | 0.233 |
| Malignant Cancer, n (%) | 146 (10.02) | 61 (16.76) | 31 (8.54) | 22 (6.06) | 32 (8.72) | **<.001** |
| AKI, n (%) | 1411 (96.84) | 352 (96.70) | 350 (96.42) | 348 (95.87) | 361 (98.37) | 0.246 |
| Sepsis, n (%) | 1403 (96.29) | 349 (95.88) | 350 (96.42) | 344 (94.77) | 360 (98.09) | 0.117 |
| Vasoactive, n (%) | 979 (67.19) | 259 (71.15) | 243 (66.94) | 222 (61.16) | 255 (69.48) | **0.024** |
| CRRT, n (%) | 358 (24.57) | 81 (22.25) | 92 (25.34) | 79 (21.76) | 106 (28.88) | 0.094 |
| Antibiotics, n (%) | 1403 (96.29) | 349 (95.88) | 350 (96.42) | 344 (94.77) | 360 (98.09) | 0.117 |
| Los Hospital, days | 24.19 (16.71, 37.08) | 21.37 (14.91,31.01) | 23.12 (16.16,33.57) | 24.80 (16.55,39.67) | 29.04 (20.17,43.54) | **<.001** |
| Los ICU, days | 16.82 (10.09, 25.68) | 13.40 (8.17,20.34) | 16.10 (10.48,24.11) | 16.90 (9.84,26.46) | 21.08 (12.94,31.48) | **<.001** |
| Hospital Mortality, n (%) | 385 (26.42) | 122 (33.52) | 95 (26.17) | 82 (22.59) | 86 (23.43) | **0.003** |
| ICU Mortality, n (%) | 299 (20.52) | 86 (23.63) | 74 (20.39) | 65 (17.91) | 74 (20.16) | 0.295 |
| 30-day mortality, n (%) | 345 (23.68) | 117 (32.14) | 84 (23.14) | 73 (20.11) | 71 (19.35) | **<.001** |
| 90-day mortality, n (%) | 500 (34.32) | 161 (44.23) | 120 (33.06) | 108 (29.75) | 111 (30.25) | **<.001** |

Abbreviations: VAP, Ventilator-Associated Pneumonia; PNI, Prognostic Nutritional Index; SBP, Systolic Blood Pressure; DBP, Diastolic Blood Pressure; MBP, Mean Blood Pressure; INR, International Normalized Ratio; PT, Prothrombin Time; PTT, Partial Thromboplastin Time; ALT, Alanine Aminotransferase; ALP, Alkaline Phosphatase; AST, Aspartate Aminotransferase; LDH, Lactate Dehydrogenase; BUN, Blood Urea Nitrogen; RBC, Red Blood Cell Count; RDW, Red Cell Distribution Width; WBC, White Blood Cell Count; SpO2, Oxygen Saturation; PO2, Partial Pressure of Oxygen; PCO2, Partial Pressure of Carbon Dioxide; PaO₂-FiO₂ ratio, Partial Pressure of Oxygen to Fraction of Inspired Oxygen Ratio; CCI, Charlson Comorbidity Index; APS III, Acute Physiology Score III; LODS, Logistic Organ Dysfunction System; OASIS, Oxford Acute Severity of Illness Score; SOFA, Sequential Organ Failure Assessment; SAPS II, Simplified Acute Physiology Score II; GCS, Glasgow Coma Scale; SIRS, Systemic Inflammatory Response Syndrome; CURB-65, Confusion, Urea, Respiratory Rate, Blood Pressure, Age ≥65; AKI, Acute Kidney Injury; CRRT, Continuous Renal Replacement Therapy. PNI quartiles: Quartile 1 (≤31.25), Quartile 2 (31.26–36.87), Quartile 3 (36.88–41.65), Quartile 4 (≥41.66). cases with PNI >200 were excluded from the analysis.

**Supplementary Table S2** Cox Regression of PNI and 30-/90-Day Mortality in VAP Patients (Sensitivity Analysis Excluding Extreme Outliers)

|  | Model 1 | | |  | Model 2 | | |  | Model 3 | | |
| --- | --- | --- | --- | --- | --- | --- | --- | --- | --- | --- | --- |
|  | HR (95% CI) | P value | P for trend |  | HR (95% CI) | P value | P for trend |  | HR (95% CI) | P value | P for trend |
| 30-day mortality | |  |  |  |  |  |  |  |  |  |  |
| Continuous | 0.97(0.96-0.98) | <0.001 |  |  | 0.98(0.96-0.99) | <0.001 |  |  | 0.98(0.96-0.99) | 0.003 |  |
| Q1 (Reference) |  |  | <0.001 |  |  |  | 0.001 |  |  |  | 0.004 |
| Q2 | 0.68(0.51-0.9) | 0.006 |  |  | 0.65(0.49-0.87) | 0.003 |  |  | 0.67(0.5-0.89) | 0.006 |  |
| Q3 | 0.58(0.44-0.78) | <0.001 |  |  | 0.66(0.49-0.88) | 0.006 |  |  | 0.69(0.51-0.93) | 0.016 |  |
| Q4 | 0.55(0.4-0.73) | <0.001 |  |  | 0.61(0.45-0.82) | 0.001 |  |  | 0.62(0.46-0.85) | 0.003 |  |
| 90-day mortality |  |  |  |  |  |  |  |  |  |  |  |
| Continuous | 0.97(0.96-0.98) | <0.001 |  |  | 0.98(0.97-0.99) | <0.001 |  |  | 0.98(0.97-0.99) | <0.001 |  |
| Q1 (Reference) |  |  | <0.001 |  |  |  | 0.002 |  |  |  | 0.003 |
| Q2 | 0.68(0.54-0.86) | 0.002 |  |  | 0.67(0.53-0.85) | <0.001 |  |  | 0.65(0.51-0.83) | <0.001 |  |
| Q3 | 0.6(0.47-0.77) | <0.001 |  |  | 0.68(0.53-0.88) | 0.003 |  |  | 0.69(0.53-0.89) | 0.004 |  |
| Q4 | 0.6(0.47-0.77) | <0.001 |  |  | 0.67(0.52-0.86) | 0.002 |  |  | 0.66(0.51-0.85) | 0.002 |  |

Model 1, unadjusted. Model 2, covariates including age, congestive heart failure, cerebrovascular disease, chronic pulmonary disease, liver disease, renal disease, malignant cancer, CRRT, were adjusted. Model 3, covariates including age, congestive heart failure, cerebrovascular disease, chronic pulmonary disease, liver disease, renal disease, malignant cancer, CRRT, INR, PT, PTT, BUN, creatinine, glucose, sodium, chloride, AST, LDH, total bilirubin, SOFA, CCI, CURB-65, were adjusted. PNI quartiles: Quartile 1 (≤31.25), Quartile 2 (31.26–36.87), Quartile 3 (36.88–41.65), Quartile 4 (≥41.66). Cases with PNI >200 were excluded from the analysis.


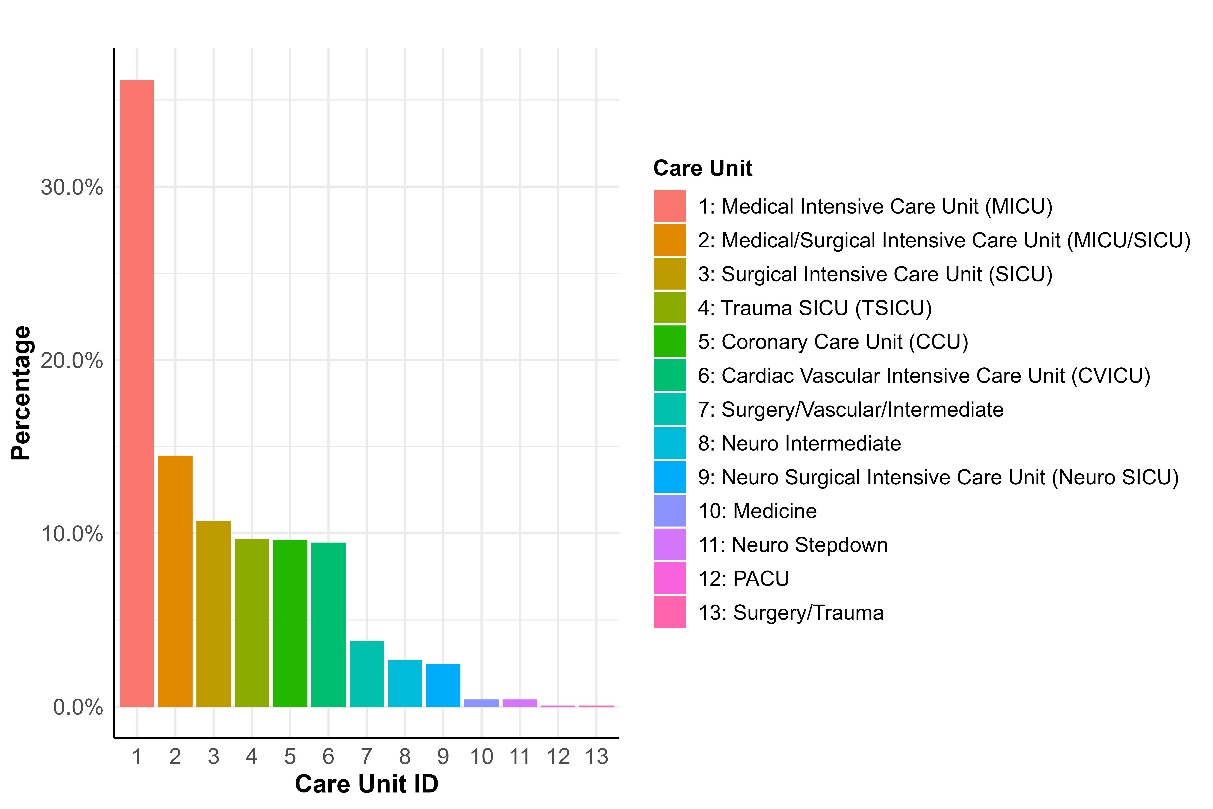


**Supplementary Figure 2** Distribution of First ICU Care Units in the Study Cohort.


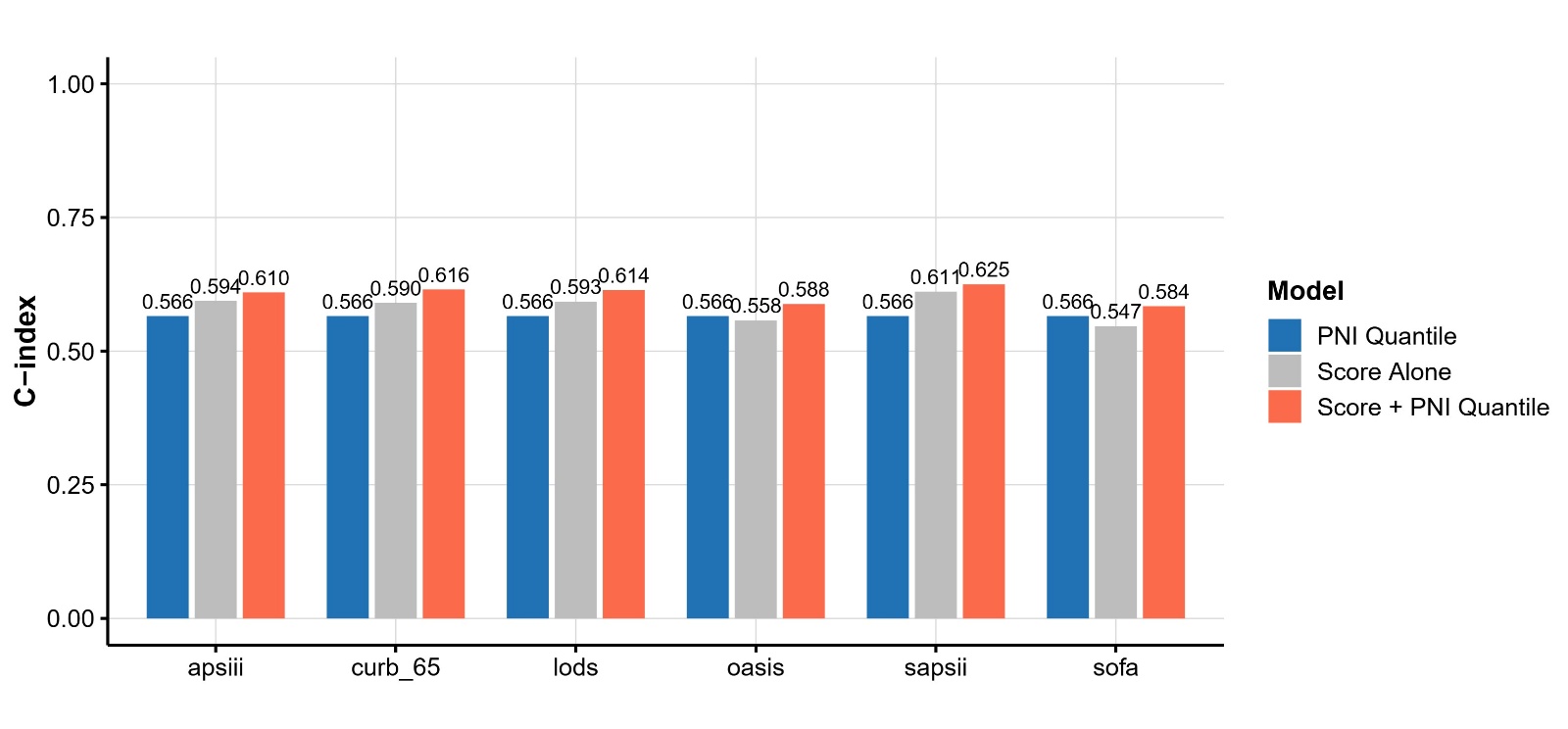


**Supplementary Figure 3** C-index comparison of PNI Quartiles, clinical scores, and combined models for 30-day mortality prediction. PNI quartiles: Quartile 1 (≤31.25), Quartile 2 (31.26–36.87), Quartile 3 (36.88–41.65), Quartile 4 (≥41.66). cases with PNI >200 were excluded from the analysis.


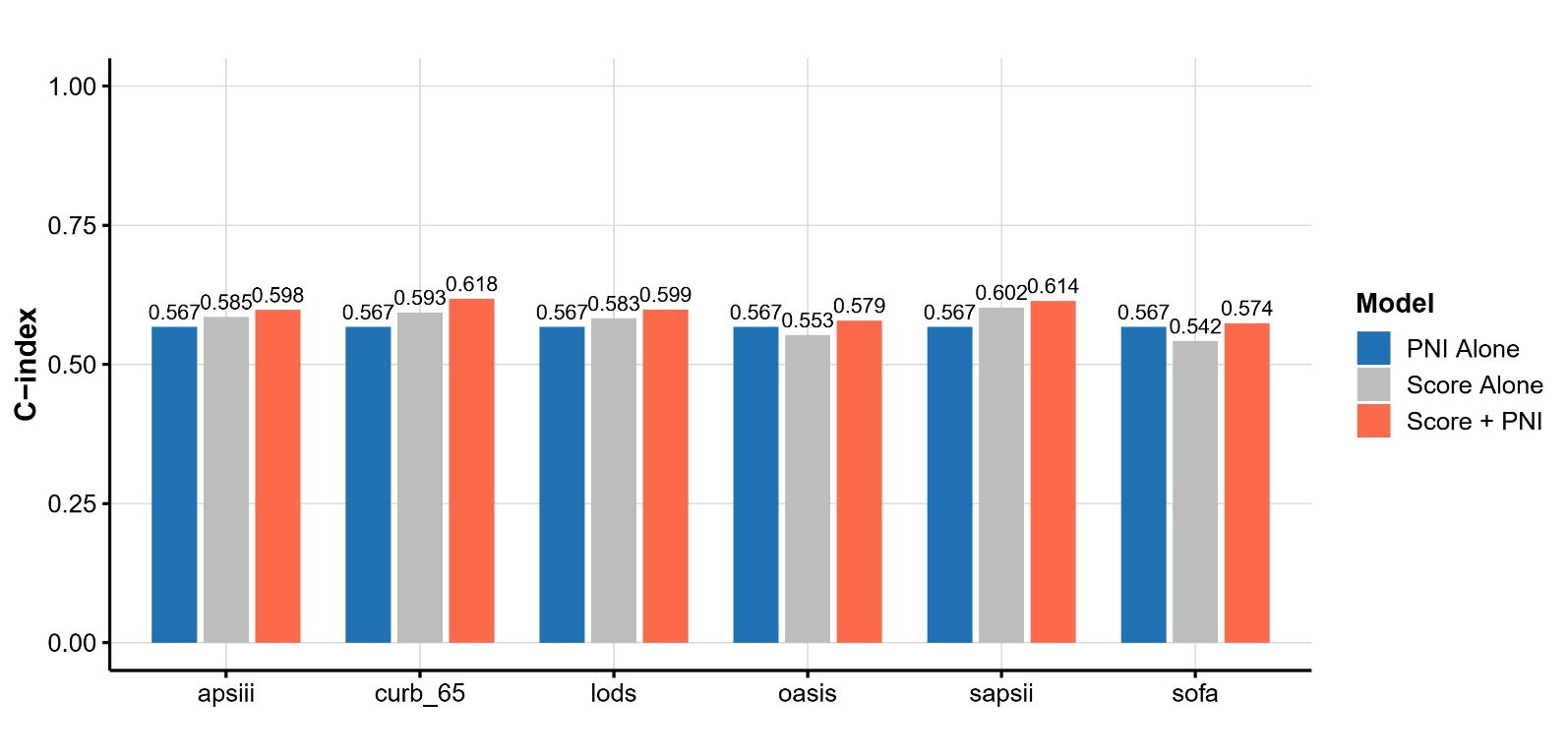


**Supplementary Figure 4** C-index comparison of PNI, clinical scores, and combined models for 90-day mortality prediction. PNI quartiles: Quartile 1 (≤31.25), Quartile 2 (31.26–36.87), Quartile 3 (36.88–41.65), Quartile 4 (≥41.66). cases with PNI >200 were excluded from the analysis.


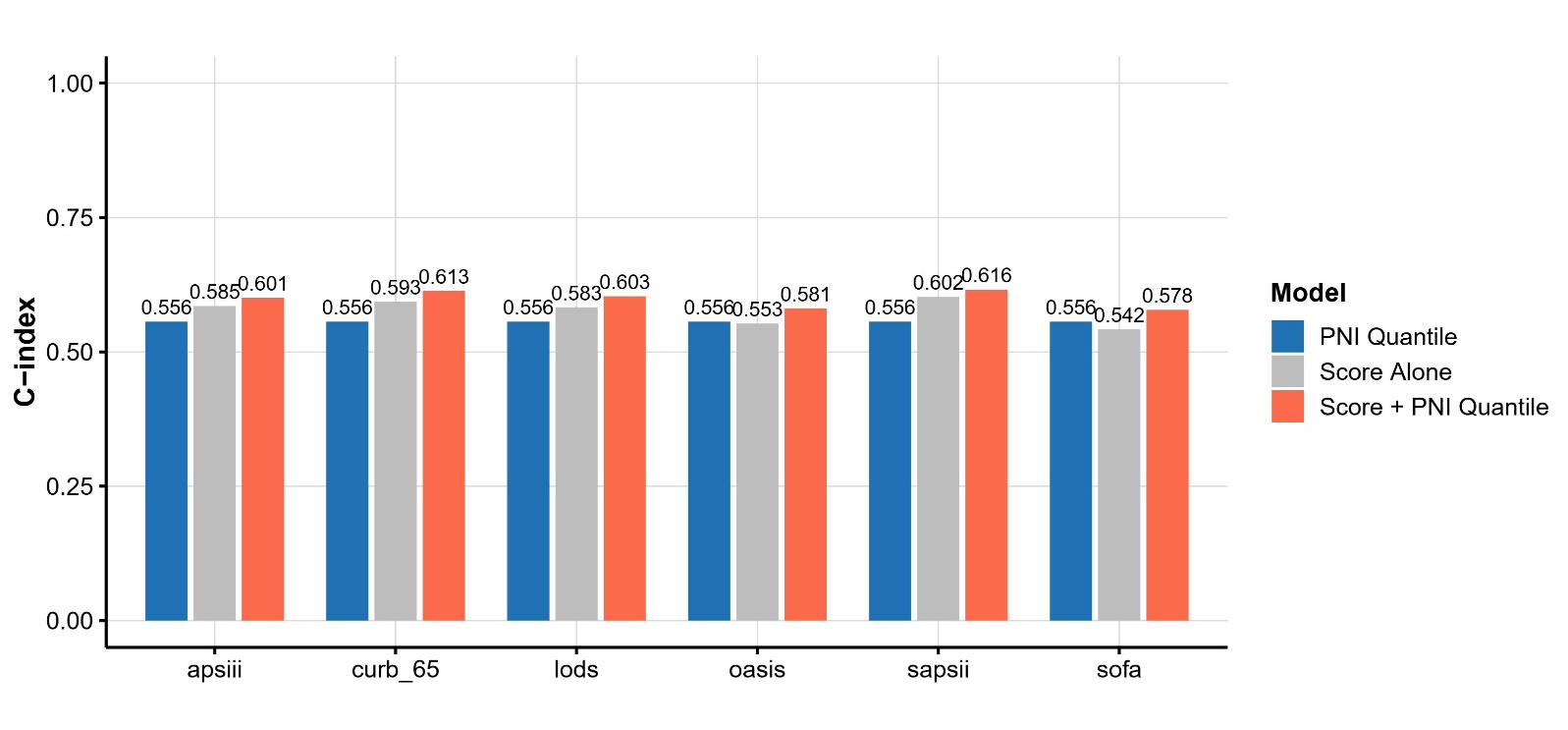


**Supplementary Figure 5** C-index comparison of PNI Quartiles, clinical scores, and combined models for 90-day mortality prediction. PNI quartiles: Quartile 1 (≤31.25), Quartile 2 (31.26–36.87), Quartile 3 (36.88–41.65), Quartile 4 (≥41.66). cases with PNI >200 were excluded from the analysis.
